# Mechanisms of Phenol Adsorption on Banana Leaves and Coffee Husk Biochars

**DOI:** 10.1021/acsomega.4c07665

**Published:** 2025-04-16

**Authors:** Melany Alejandra Ruiz Lopez, Guilherme Max Dias Ferreira, Matheus Torres Duarte Figueiredo, Gabriel Max Dias Ferreira, José Romão Franca, Evanise da Silva Penido, Jenaina Ribeiro Soares, Raphael Longuinhos Monteiro Lobato, Aparecida Barbosa Mageste

**Affiliations:** a Laboratory of Physical Chemistry and Environmental Chemistry, Department of Chemistry, Federal University of Ouro Preto, Campus Morro do Cruzeiro, Ouro Preto, MG 35400-000, Brazil; b Department of Chemistry, Institute of Natural Sciences, Federal University of Lavras, Campus Universitário, Lavras, PO Box 3037, Minas Gerais 37200-000, Brazil; c Department of Physics, Federal University of Lavras, Campus Universitário, Lavras, Minas Gerais 37200-000, Brazil

## Abstract

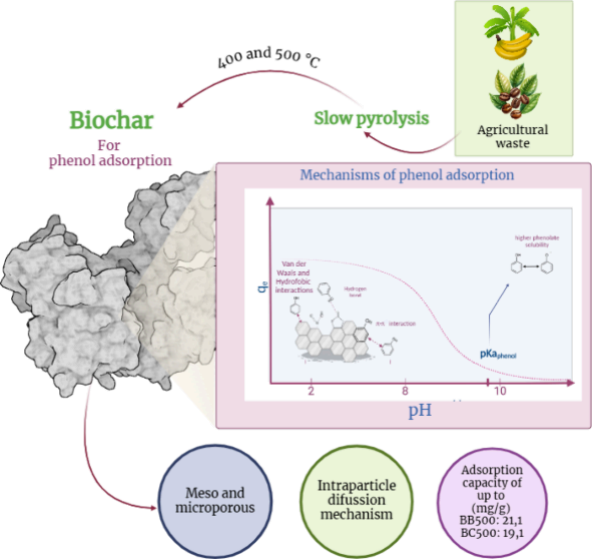

In this study, biochars were produced from banana leaves (BB) and coffee husk (BC) for phenol adsorption. The biochars were characterized using Fourier transform infrared spectroscopy, X-ray photoelectron spectroscopy, Raman spectroscopy, textural analysis, point of zero charge measurement, and determination of surface acidic and basic groups. For both biochars, a higher pyrolysis temperature led to losses of oxygenated groups as well as increases of graphitic structures and greater basic character. For biochars produced at 400 °C, phenol adsorption kinetics was best described by the pseudo-second-order model. Chemisorption involving π–π interactions was identified as the main adsorption mechanism. For biochars produced at 500 °C, a smaller pore size resulted in limited adsorption by intraparticle diffusion. The Freundlich model provided the best fit to the isotherm data due to the high surface heterogeneity. Moreover, the results also suggested the formation of multilayers or pore filling as adsorption mechanisms for the obtained biochars. The maximum adsorption capacity values (*q*_e_) were 13.8 and 21.2 mg g^–1^ for phenol adsorption on BB400 and BB500, and 17.3 and 19.1 mg g^–1^ for BC400 and BC500, respectively. The results showed that the agroindustrial residues are suitable for phenol adsorption in aqueous solutions.

## Introduction

Phenol is one of the most important organic precursors employed in processes to produce resins, plastics, paints, fertilizers, explosives, cosmetics, disinfectants, drugs, and other substances.^1,2^ The phenol market has been experiencing growth since the last century and is expected to continue expanding until 2024, with a projected increase of 4.36% over the 11 million tons produced in 2023.^3,4^ Given this level of production, there are concerns about the quantities of phenol discharges into the environment from the pharmaceutical, plastic, paper, petrochemical, and oil refinery industries, which generate effluents containing phenol at levels ranging from 0.1 to 30,000 mg L^–1^.^2,5^ Phenol is also one of the most common organic compounds detected in surface waters, reaching concentrations of up to 1.3 μg L^–1^.^6^ This poses risks to aquatic environments and human health due to its high toxicity, activity, and persistence.^1,6^ The limit concentration for phenol in drinking water is 1 mg L^–1^ according to the World Health Organization (WHO) recommendation. Nonetheless, the United States Environmental Protection Agency (US-EPA) has considered that a lifetime exposure of 2 mg L^–1^ of phenol is expected to not produce adverse effects.^7,8^ Therefore, it is necessary to identify and remove phenol from watercourses, as well as treat effluents contaminated by this compound to prevent its release into surface waters.

Adsorption is one of the most used processes for the removal of phenol from aqueous matrices due to its practicality, low cost, and environmentally friendly characteristics.^9^ Among various adsorbent materials used for this purpose, activated carbon stands out for its excellent adsorption capacity (Table S1, in the Supporting Information). Despite this, activated carbon is often expensive to produce and difficult to reuse at industrial scale.^10^ Thus, studies focusing on the development of alternative carbonaceous adsorbent materials are of great importance.^11^ In this scenario, biochars obtained from pyrolysis of biomass residues have emerged as sustainable materials for adsorption of phenolic compounds.^12−15^

Biochars are stable materials characterized by high fixed carbon contents and surface functional groups that enhance adsorption processes and, consequently, improve the efficient removal of pollutants.^16^ This approach provides an environmentally friendly and sustainable solution for utilizing agricultural waste. It not only addresses the disposal challenges posed by toxic waste materials but also offers significant environmental benefits. Recent studies have shown that biochar production can reduce CO_2_ emissions, contributing to global warming mitigation and, in some cases, achieving carbon neutrality.^17,18^

Several agricultural residues have already been used as precursors to produce biochars for phenol adsorption from aqueous matrices, including olive oil waste, pine fruit shells, banana peels, and pomelo peels.^19−22^ The adsorption performances observed for these biochars strongly depend on the type of biomass, the pyrolysis process parameters, and the activation methodologies used to increase biochar surface area.^13,15^ Nevertheless, biochars without any activation processes or treatments can exhibit adsorption performance comparable to that of activated or pretreated materials due to their surface properties, particularly the presence of functional groups that interact with specific adsorbates.^20,23^ This is desirable for economic and sustainable production because some activation methods use high-cost reagents and generate dangerous and/or toxic residues.^24^ Therefore, the evaluation of biomass with different lignocellulosic compositions is important for producing biochars with improved adsorption performance, especially for phenol removal.

In Brazil, coffee and banana productions are important agroindustrial sectors that generate large amounts of waste suitable for use as renewable raw materials in the production of biochars. Coffee is one of the most consumed beverages worldwide (8 million tons year^−1^),^25^ with 2 tons of waste produced per ton of grains.^26^ In 2022, worldwide coffee consumption was estimated at 168.2 million 60 kg bags.^27^ Around 80% (by mass) of coffee byproducts, including mucilage, husks, and pulp, are often discarded.^26,28^ The husks constitute the largest fraction of solid waste (43%), representing a substantial environmental threat not only due to slow degradation but also due to high concentrations of tannins, caffeine, and phenols, making the waste potentially toxic.^26,28^ Although recent studies have explored these residues for antibacterial or antioxidant applications,^29,30^ coffee residues are primarily used in animal feed.^31^ Consequently, various alternatives have emerged for obtaining byproducts such as biodiesel, biogas, catalysts, adsorbents, and even dyes, promoting green alternatives for agroindustrial residue utilization in coffee cultivation.^31,32^

Banana cultivation is another activity that generates substantial quantities of agroindustrial byproducts. Banana plants grow over periods of 8–9 months with high levels of leaf production. After fruit harvesting, plants continue to produce biomass for at least 2 more years.^33^ For each ton of bananas produced, around 4 tons of plant biomass are generated. Without specific treatment or suitable disposal, the decomposition of this material contributes to carbon dioxide emissions.^34^ Both banana leaves and coffee residues have traditionally been used as feed for ruminants to improve digestion and as packaging for artisanal food, especially in rural areas.^35^ However, the potential of banana leaves for extracting dietary fiber, lignin, antioxidants, and pigments renders them valuable waste material.^35,36^ This waste can be repurposed into environmentally beneficial products such as biochar for wastewater treatment.

Considering the problems associated with biomass byproduct generation during banana and coffee cultivation, using these byproducts for biochar production is an attractive approach. This contributes to converting organic matter into a stable form of carbon for environmental applications. In this context, this study aimed to investigate the use of biochar derived from coffee husks (CH) and banana leaves (BL) for phenol removal from aqueous solutions. In addition to the type of biomass, the effect of the pyrolysis temperature on biochar properties and adsorption capacity was evaluated. Biochars were produced at 400 (BB400 and BC400) and 500 °C (BB500 and BC500) via slow pyrolysis and characterized using Fourier transform infrared spectroscopy (FTIR), nitrogen adsorption–desorption analysis, Raman spectroscopy, scanning electron microscopy (SEM), energy-dispersive X-ray spectroscopy (EDS), X-ray photoelectron spectroscopy (XPS), acid and basic functional group analysis, and pH of point zero charge (pH_PZC_) measurements. These characterization techniques, combined with adsorption studies (kinetics and isotherms), were employed to elucidate the primary adsorption mechanisms.

## Results and Discussion

### Biochar Production Yields

The average yields of biochar production after pyrolysis of the biomasses were 38.8 and 31.3% for BB400 and BB500 obtained from BL, respectively, and 32.9 and 31.5% for BC400 and BC500 obtained from CH, respectively. These results indicated that higher final pyrolysis temperatures led to lower yields due to a greater release of volatile components during thermal degradation. The yields of the biochars obtained at a final pyrolysis temperature of 400 °C also depended on the precursor raw material, which can be attributed to the different biomass compositions, particularly their varying hemicellulose/cellulose/lignin mass ratios.^24^ BL is mainly composed of cellulose and lignin,^38,39^ while CH has lower lignin and hemicellulose contents and a high cellulose content.^40^ A higher lignin content is usually associated with greater thermal stability of the biomass because lignin degradation occurs above 500 °C,^52^ which could explain the highest yield observed for the banana leaf biochar produced at 400 °C. However, when the final temperature was increased to 500 °C, the yields were similar for both studied biomasses due to greater degradation of the lignin present in the BL, while CH showed no significant additional mass loss at the higher final temperature.

### Biochar Characterization

#### Fourier Transform Infrared Spectroscopy

**Figure 1:**
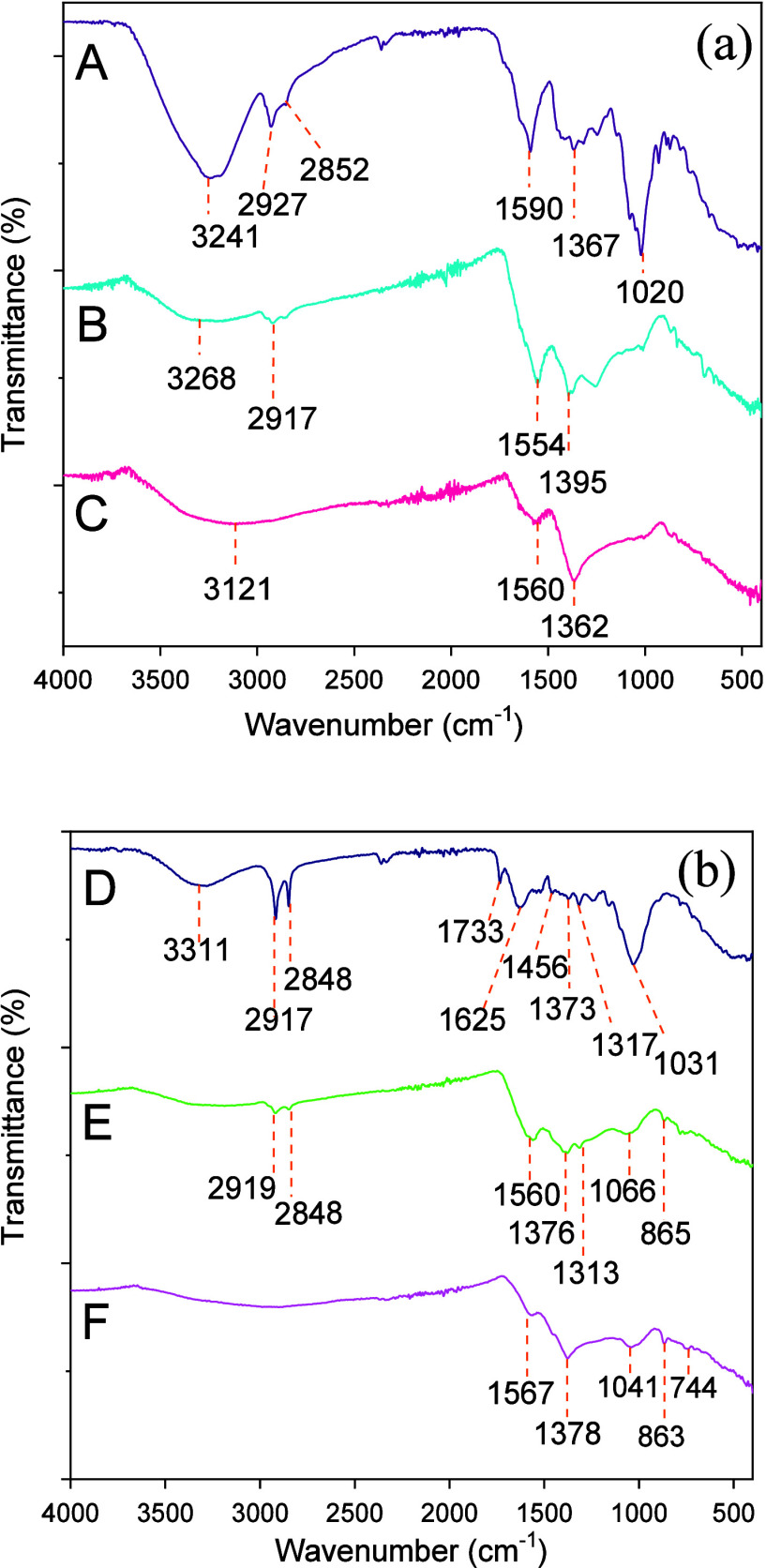
FTIR spectra of the biochars and their respective precursor biomasses: (a) CH (A), BC400 (B), and BC500 (C); (b) BL (D), BB400 (E), and BB500 (F).

FTIR spectra of the biochars were obtained to gather information about the functional groups present on the surfaces of these materials. Figure 1 shows the FTIR spectra for the produced biochars and their respective precursor biomasses.

The spectrum for the CH biomass (Figure 1a) showed a broad band at around 3241 cm^–1^ attributed to the stretching of the O–H bond.^42^ The relative intensity of this band decreased after pyrolysis, indicating a loss of hydroxyl groups during thermal decomposition. Bands at 2927 and 2852 cm^–1^ in the biomass spectrum and at 2927 cm^–1^ in the BC400 spectrum were associated with the symmetric and asymmetric stretching of the C–H bonds in the aliphatic chains of nondegraded lignocellulosic compounds.^43^ Bands at 1367 cm^–1^ in the CH biomass spectrum and at 1395 and 1362 cm^–1^ in the BC400 and BC500 spectra, respectively, could be attributed to the bending of the C–H or O–H bonds.^44^ In general, these bands were of low relative intensity in the biochar spectra (and absent in BC500), suggesting the transformation of the aliphatic chains of lignocellulosic compounds into aromatic or graphitic structures.^45^ Bands at 1247, 1145, 1020, and 845 cm^–1^ in the CH biomass spectrum were assigned to the symmetric and asymmetric stretching of C–O–C bonds in dialkyl and aryl ethers or C–O bonds in alcohols.^46^ These bands were of low intensity in the BC400 spectrum and nearly disappeared in the BC500 spectrum, in which only the band at 1020 cm^–1^ was observed. This could suggest that only more stabilized C–O oxygenated groups were retained at the highest pyrolysis temperature, as discussed in X-ray photoelectron spectroscopy section. Finally, bands corresponding to the stretching of the C=O bond of the carboxylate group or aromatic C=C bonds were observed at 1590, 1554, and 1560 cm^–1^^38^ in the spectra of the CH biomass, BC400, and BC500, respectively.

Most of the bands present in the spectra of the BL biomass and the corresponding biochars (Figure 1b) appeared at positions similar to those observed in the spectra of the CH materials (Figure 1a). A band at 3311 cm^–1^, corresponding to the symmetric stretching of the O–H bond, was almost absent in the biochar spectra. Bands associated with the symmetric and asymmetric stretching of C–H bonds in methylene and methyl groups were observed at 2917 and 2848 cm^–1^ in the biomass spectrum, respectively.^23,47,48^ These bands decreased in relative intensity in the BB400 spectrum and disappeared in the BB500 spectrum. Bands corresponding to the bending of C–H or O–H bonds were present at 1456 and 1373 cm^–1^ in the biomass spectrum^44^ and appeared at 1376 and 1378 cm^–1^ in the BB400 and BB500 spectra. In the biochar spectra, these bands may overlap with those associated with symmetric stretching of the carboxylate group. Bands at 1733 and 1625 cm^–1^ in the biomass spectrum were attributed to the asymmetric stretching of carbonyl groups in ketone and ester functions of quinone or lactone, respectively, from the nondegraded lignin present in BL.^49,50^ These bands disappeared in the biochar spectra, where bands at 1560 and 1567 cm^–1^ for BB400 and BB500, respectively, were observed, probably corresponding to the asymmetrical stretching of the C=O bond of the carboxylate group.^49,50^ Finally, bands associated with the symmetric and asymmetric stretching of the C–O–C group in dialkyl and aryl ethers were present at 1246, 1160, and 1031 cm^–1^ in the BL biomass spectrum but were of low relative intensity or absent in the biochar spectra.

In summary, the FTIR results revealed that for both biomasses pyrolysis led to the loss of some oxygenated functional groups. This effect was more pronounced at 500 °C, indicating the elimination of volatile compounds derived from the decomposition of lignocellulosic compounds.^51^ Additionally, the pyrolysis of the studied biomasses led to transformations in the aliphatic chains of such compounds, which may have promoted graphitization of the materials, especially at higher temperatures, as evidenced in the next sections.

#### Raman Spectroscopy

Raman spectra of the biochars were obtained to confirm the effect of the pyrolysis temperature on the aromaticity and crystallinity of the biochars produced. Figure S1 (Supporting Information) shows the Raman spectra of the biochars and their fitting with Lorentz functions used for the crystallite size (*L*_a_) estimation. The *L*_a_ values of each sample are presented in Figure S2 (Supporting Information).

The Raman spectra of the biochars showed broad peaks in two regions close to 1358 cm^–1^ (D band) and 1593 cm^–1^ (G band). The D band is generally associated with vibrations of aromatic C structures, commonly observed in materials with a higher degree of disorder, in contrast to the G band, which corresponds to the symmetrical elongation of C–C bonds typical of several carbonaceous materials.

The results showed that BC400 and BB400 samples had smaller crystallite sizes compared with BC500 and BB500 samples. This can be associated with the formation of more organized carbonaceous structures during the pyrolysis process at higher temperatures. Variations in the values of *L*_a_ of materials with carbonaceous structures indicate different levels of graphitization. Thus, materials with more organized structures suggest a predominance of sp^2^ domains and reflect higher *L*_a_ values. In the case of samples produced at 500 °C, a difference in the crystallite sizes was noted. These changes could be explained by bond rupture and the formation of more complex carbon–carbon bonds, primarily involving the reduction of cellulose and hemicellulose, which is present in higher proportions in the CH biomass,^52^ as suggested by the FTIR spectra, resulting in a lower *L*_a_ for BC500 compared to BB500. In summary, the Raman measurements showed that BC500 and BB500 have larger crystallite sizes, indicating a more ordered structure compared with BC400 and BB400, which have a more amorphous structure. This highlights the dependence of the biochar structure on the pyrolysis temperature.

#### X-ray Photoelectron Spectroscopy

**Figure 2:**
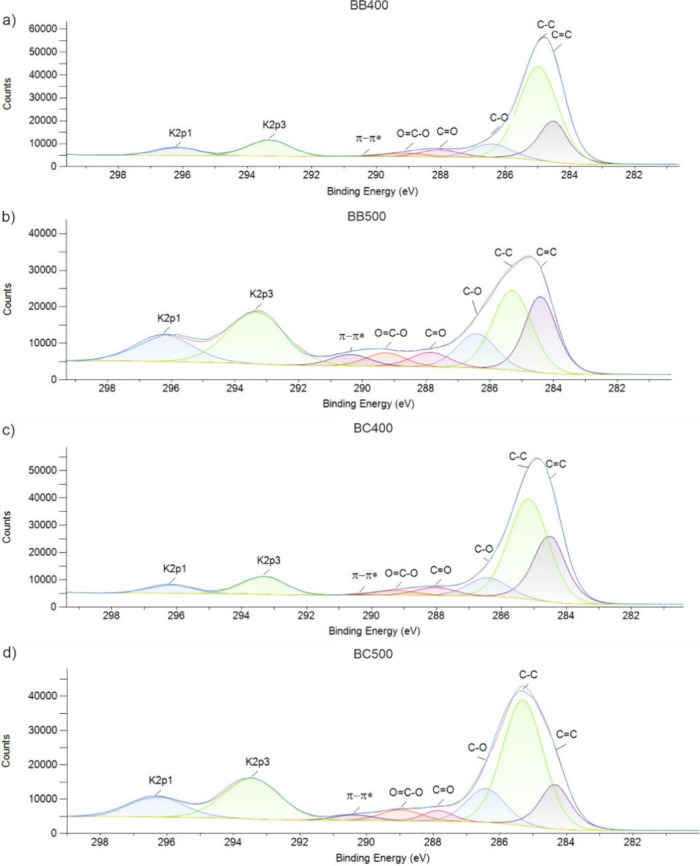
High-resolution C 1s XPS spectra of biochars derived from (a) BB400, (b) BB500, (c) BC400, and (d) BC500.

Figure 2 shows the XPS high-resolution spectra for BB400, BC400, BB500, and BC500. The atomic percentages of functional groups in biochars are given in Table 1. The survey spectra were acquired (not shown here), which demonstrate that the most relevant peak for this discussion is the high-resolution spectrum focused on the C 1s peak, typically found in the 283–285 eV range, and neighboring peaks. The sp^2^ hybridized carbon (C=C, ∼284.5 eV) was identified as an indicator of aromatic or graphitic structures, while the sp^3^ hybridized carbon (C–C, ∼285.0 eV) was attributed to aliphatic carbon. Oxygen-containing functional groups were detected, especially C–O (∼286.49 eV), assigned to alcohols, ethers, or phenols; C=O (∼287.89 eV), associated with ketones, aldehydes, or lactones; and O=C–O (∼288.79 eV), corresponding to carboxylates, esters, or anhydrides. Additionally, a π–π* satellite peak (∼290.67 eV) was identified, indicative of electron delocalization in conjugated aromatic systems. Lastly, potassium-related signals were detected at K 2p3 (∼293.30 eV) and K 2p1 (∼296.10 eV).^53,54^

**Table 1:** Atomic Percentage of Functional Groups in Biochars before (BB400, BB500, BC400, and BC500) and after Phenol Adsorption (BB400F, BB500F, BC400F, and BC500F) in the C 1s Region XPS Spectra

	C=C	C–C	C–O	C=O	O=C–O	π–π*	K 2p
**sample**	**(%)**						
BB400	21.03	59.53	8.61	4.55	2.34	0.42	3.51
BB500	26.55	32.79	14.15	6.02	5.58	4.67	10.24
BC400	28.94	51.52	9.52	4.13	2.45	0.24	3.21
BC500	15.27	53.10	13.56	3.37	4.73	2.37	7.60
BB400F	47.75	35.81	8.62	4.11	2.89	0.59	0.23
BB500F	34.93	43.62	9.66	5.22	3.43	2.10	1.04
BC400F	19.77	60.00	11.02	3.94	3.75	1.17	0.35
BC500F	46.14	35.87	7.48	5.50	3.39	0.65	0.97

The XPS analysis confirms a trend of increased graphitization in BB500 produced at a higher pyrolysis temperature. The C 1s spectra show a greater contribution of sp^2^ carbon (C=C, ∼284.5 eV) in BB500 when compared with BB400, indicating increased aromaticity and graphitic character. This trend aligns with FTIR results, in which the aromatic C=C stretching band is enhanced after pyrolysis. In contrast, the intensity of C–C (∼285 eV, sp^3^ carbon) is higher in BB400, corroborating the presence of more disordered or aliphatic structures.

For BC500, the XPS data indicate an apparent contrary trend, showing a reduction in C=C compared to BC400. This suggests that the structural transformation of the biochar during pyrolysis is influenced by the nature of the precursor material (coffee husks *versus* banana leaves). Coffee husks contain more hemicellulose and extractives, which could lead to different decomposition pathways that do not favor graphitization as efficiently as in banana leaves biochar, which corroborate higher *L*_a_ values observed for BB500 compared with BC500 from Raman analysis. On the other hand, at moderate pyrolysis temperatures (400–500 °C) biochars undergo multiple competing reactions, including aromatization, cross-linking, and deoxygenation.^55^ However, if the structural rearrangement is not sufficiently efficient, a fraction of the biochar at 500 °C can contain more disordered carbon than at 400 °C, leading to an apparent reduction in the C=C signal in BC500. This is particularly relevant for biomass sources with a higher oxygen or heteroatom content, as incomplete condensation may result in disordered carbon structures rather than well-ordered graphitic domains.

While the expected graphitization trend suggests an increase in C=C content, the persistence of oxygen-containing functional groups (C–O, C=O, and O=C–O) in BC500 may influence peak assignment in the XPS spectra. The binding energy of oxygen-functionalized carbon can overlap with sp^2^ carbon, making the deconvolution process more complex. If some C=C sites remain functionalized, then they might be underestimated in peak fitting, leading to a lower apparent sp^2^ contribution in BC500 than in BC400. The π–π* satellite peak (∼290.7 eV) further supports increased aromaticity at 500 °C (corroborating Raman spectroscopic analyses), reflecting the greater degree of conjugation and electron delocalization within the carbon matrix for BC500 and BB500.

In a more detailed analysis of the content of oxygen-containing functional groups, XPS analyses detected the highest percentage of oxygen functional groups on biochars produced at 500 °C, mainly in the form of C–O (∼286.5 eV), C=O (∼287.9 eV), and C=C–O (∼288.8 eV). However, the FTIR spectra suggested that the content of −OH and C–O–C groups decreases with increasing pyrolysis temperature. These groups may not be newly formed during pyrolysis but rather represent the fraction of oxygen functional groups that persist despite high-temperature treatment. For example, the alkali metal K, present in high content for biochars obtained at 500 °C (see EDS results) can enhance oxygen functional groups retention, with high char conversion.^56^

#### pH_PZC_ and Contents of Acidic and Basic Groups

The data used to determine the pH_PZC_ and the conductometric titration curves for the determination of the acidic and basic group contents in the CH and BL biochars are showed in Figure S3 (Supporting Information). The pH_PZC_ values and contents of acidic and basic functions (N) are showed in Table 2.

**Table 2:** pH_PZC_ Values and Contents of Acidic and Basic Groups in the Biochars of CH (BC400 and BC500) and BL (BB400 and BB500)

adsorbent	pH_PZC_	*N*_acidic functions_ (mmol g^–1^)	*N*_basic functions_ (mmol g^–1^)
BC400	10.3	2.09	1.57
BC500	10.3	1.86	1.18
BB400	10.0	1.57	2.64
BB500	10.6	1.63	3.18

The pH_PZC_ values were in agreement with those reported in the literature for biochars obtained from similar sources.^57,58^ For all materials, these values were in the basic region. For the BL biochars, the higher pyrolysis temperature slightly increased the pH_PZC_. It suggests that groups with higher p*K*_a_ (basic character) were proportionally more retained during the pyrolysis process when compared with the biochar obtained at a lower temperature (BB400). Furthermore, functional groups with lower p*K*_a_ (acidic character) were proportionally volatilized to a greater extent.. This hypothesis was supported by the higher content of basic groups in the BL biochar obtained at the higher pyrolysis temperature, with a small change in the quantity of acidic functions. For the CH biochars, a higher pyrolysis temperature decreased the quantities of both acidic and basic functions.

#### Surface Area, Pore Volume, and Pore Size of the Biochars

Determinations of specific surface area, pore volume, and pore size of the biochars were performed (Table S2, in the Supporting Information). The CH biochars were characterized as mesoporous, having a smaller pore volume and larger pore size compared with the BL biochars. The specific surface area of the CH biochars slightly increased (almost an insignificant change considering the precision of the measurements) with an increase in the pyrolysis temperature. Furthermore, larger pore volumes and a smaller pore sizes were observed. In contrast, for the BL biochars, a higher pyrolysis temperature resulted in slightly smaller specific surface areas with decreases in both the volume and size of the pores, which are classified as micropores.

Studies reported in the literature have found that an increase in pyrolysis temperature usually leads to an increase in the specific surface area and pore volume of biochar.^59,60^ This is due to carbonization processes that cause volatilization of organic matter from the biomass through the rupture of chemical bonds. It leads to the formation of defects or tubular structures, resulting in the formation of micropores and mesopores.^61^ The results suggested that at the higher pyrolysis temperature, the volatile compounds present in the CH biochars were more rapidly eliminated or condensed. Diffusion from the interior to the surface of the biochar could have contributed to the formation of larger pores^62^ when compared with those in the BL biochars.

The specific surface area of biochar can reach a maximum value at a certain pyrolysis temperature, subsequently decreasing with a further increase in temperature,^62,63^ mainly due to the formation of substantial quantities of ash, which can block the biochar pores.^64,65^ Factors that influence the specific surface area of biochar include the amount of volatile material and biochar yield, both of which have an inverse relationship with the specific surface area.^61^ This was observed for the CH biochars, where the BC400 showed a higher yield and a lower specific surface area than BC500.

The lower surface area of BB500 could be explained by the high volatile material content of BL and the greater amount of ash blocking its micropores. Previous reports have indicated that for some biochars temperatures higher than 500 °C can result in the formation of nanopores, which are not quantified by nitrogen adsorption measurements, resulting in a negative artifact in the specific surface area measurement, characterizing the material as microporous.^55^

#### Scanning Electron Microscopy and Energy-Dispersive Spectroscopy

**Figure 3:**
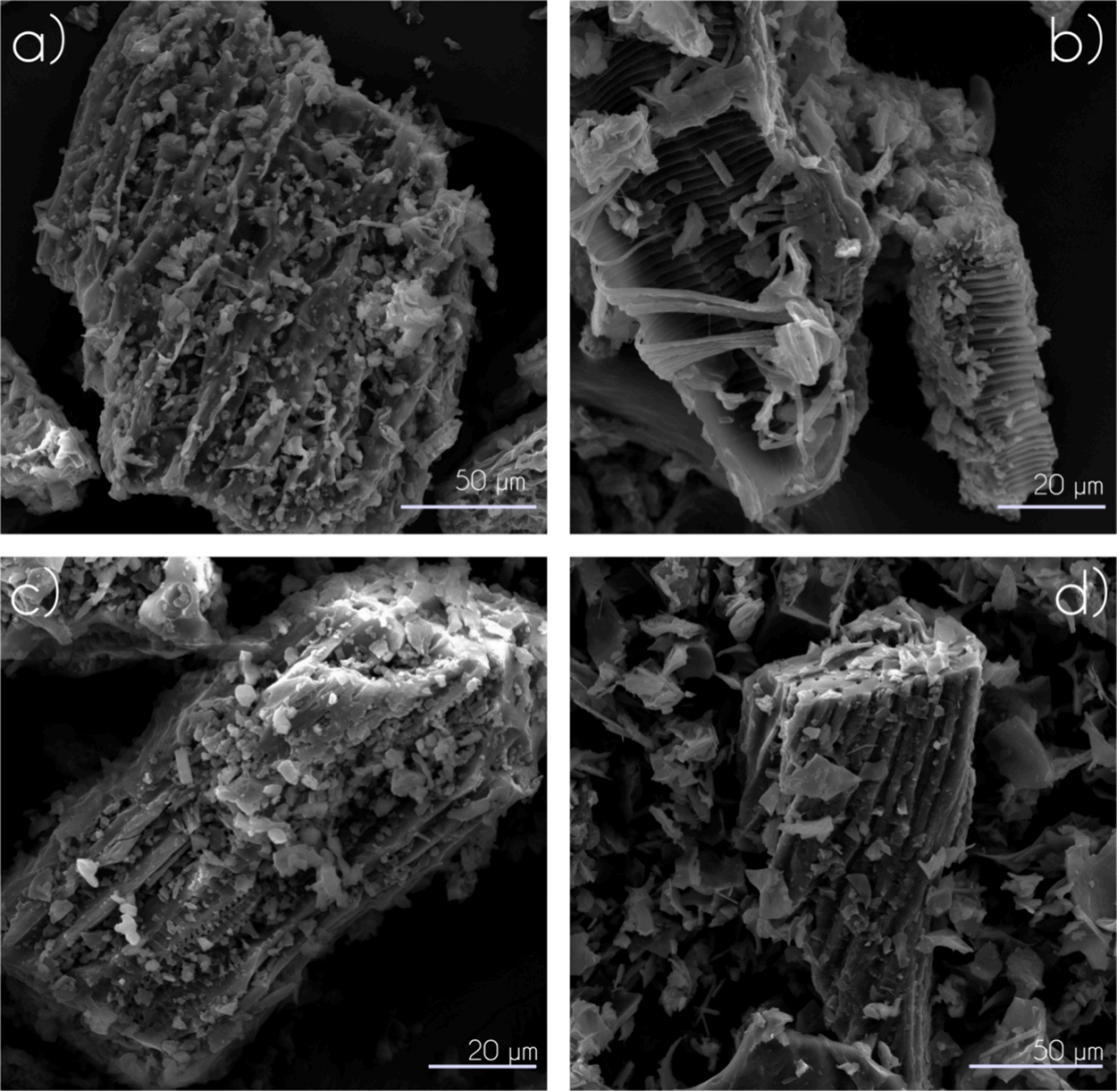
SEM micrographs of biochars (a) BB400, (b) BB500, (c) BC400, and (d) BC500.

The morphology of the biochars was analyzed using SEM micrographs (Figure 3) and EDS was used to identify the main constituent elements on the surface of the biochars (Figure S4, Supporting Information). The micrographs revealed surfaces with a high degree of defects in all biochars. In general, the analyzed samples exhibited rough surfaces with agglomerates of small particles and the presence of crater-like pores. Specifically for biochars obtained at 400 °C, a higher content of fiber-like structures remained after the pyrolysis process. In biochars produced at 500 °C, a different morphology was observed between BB and BC. In BB500 (Figure 3b), fibrous particles with organized and repeated structures along their length were observed, forming an interwoven agglomerate. It indicates a high heterogeneity in this biochar. In contrast, the surface of BC500, despite the presence of fibers from the coffee husks, exhibited less roughness, with crater-like pores and smaller deformed particles scattered around the fibers.

The EDS results (Figure S4, Supporting Information) revealed that the primary constituents of the biochars were carbon (∼50–60%), oxygen (∼30–35%), and potassium (∼8–15%). Additionally, calcium (1.5–2.4%) and magnesium (0.6–1.2%) were identified, along with trace amounts of silicon and chlorine. Increasing the pyrolysis temperature altered the proportion of these elements. For example, only magnesium, chlorine, and calcium were detected in both biochars at 500 °C, with trace amounts of silicon observed exclusively in BB500. In contrast, an increase in potassium content was observed with higher pyrolysis temperatures for both biochars. This could be associated with greater mass loss and the reduction of organic compounds in the biomass, which might enhance the basic character of the biochars, particularly for BB500.

The main minerals found in all biochars were related to the growth and development processes of the plants. For example, potassium plays a crucial role in water retention, fruit ripening, and the translocation of sugars and acids; calcium assists in nutrient retention, increases plant stability, and helps prevent bacterial and fungal attacks; and magnesium, primarily found in stems and leaves, aids in enzyme synthesis and chlorophyll production, among other functions.^66^

### Assays of Phenol Adsorption on the Biochars

#### Effect of pH

**Figure 4:**
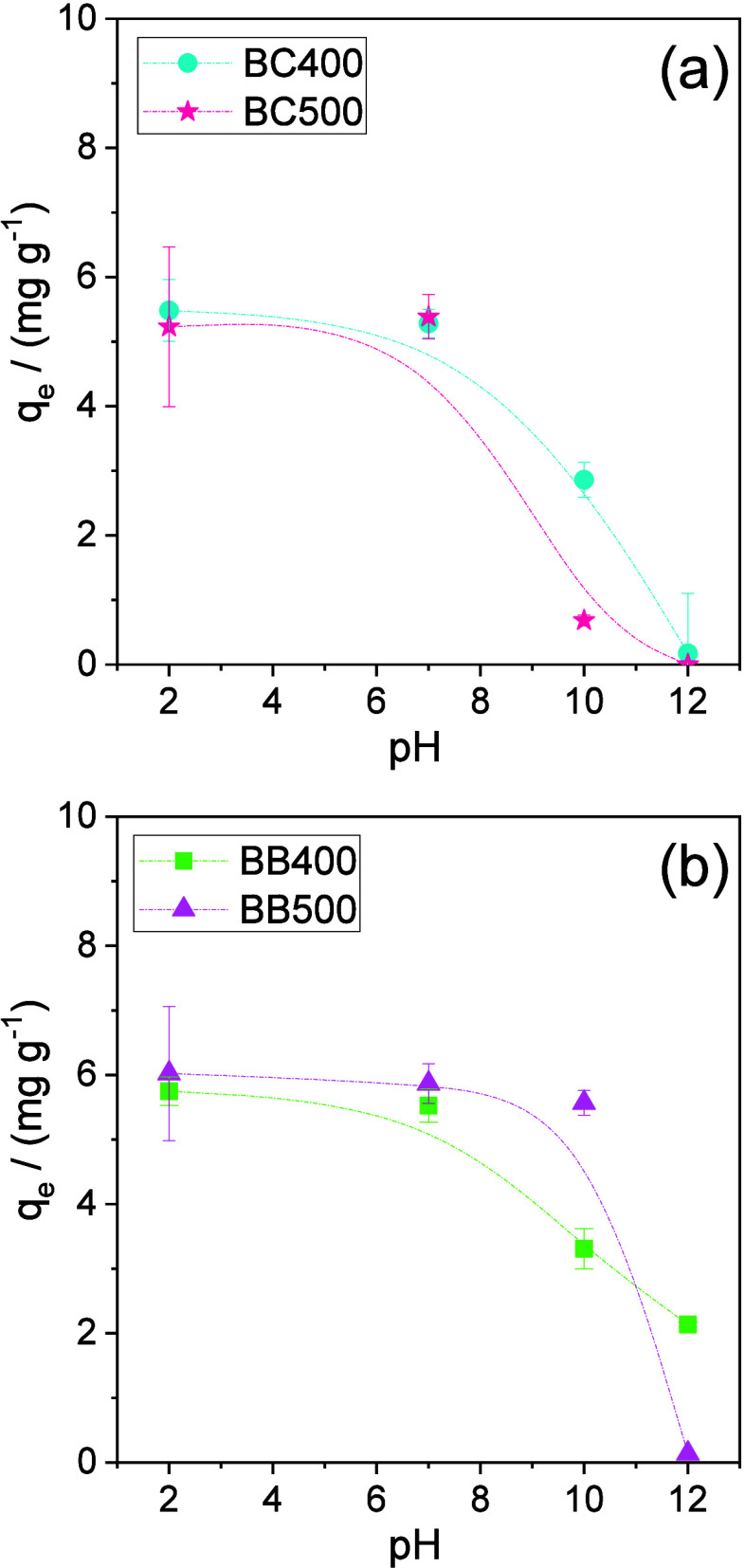
Effect of pH on the amount of phenol adsorbed by biochars obtained from (a) CH and (b) BL produced at 400 and 500 °C. Adsorption conditions: *C_i_* = 40.0 mg L^–1^, 150 rpm, 1.00 g L^–1^ of adsorbent, contact time of 22 h, and 25.0 °C. Each data point represents the mean of duplicates, with error bars indicating the standard deviation from the mean. For many data points, the standard deviation was smaller than the size of the symbol, making the error bars indistinguishable.

Both the surface charge of the biochar and the net charge of phenol depend on the pH of the medium, which can influence the electrostatic interactions between phenol and the adsorbent. Figure 4 shows the effect of the pH on phenol adsorption by the different produced biochars.

For all biochars, the amount of phenol adsorbed remained constant as the pH increased from 2.0 to 7.0. However, the *q*_e_ values decreased as the pH rose to 12.0, suggesting that electrostatic interactions influenced the adsorption process. In the case of BB500 (Figure 4b), the adsorbed amount remained constant up to pH 10.0 and then dropped to zero at pH 12.0. This indicates that specific surface groups on this biochar contributed to higher phenol adsorption at pH 10.0 through nonelectrostatic interactions compared to the other biochars.

Phenol is a weak acid (p*K*_a_ = 9.86) and predominantly carries a negative charge in the form of the phenolate ion at pH values above 10.0. This could explain the lowest *q*_e_ values at higher pH, especially at pH 12.0, since the pH_PZC_ values of the biochars were around 10. Under these conditions, surface groups on the biochars—such as lactones, carboxylic acids, and phenolics—were dissociated, creating a negative net charge on the biochar surfaces and resulting in electrostatic repulsion of the phenolate ion.^67^ Additionally, the phenolate ion exhibits higher solubility in basic solutions, leading to stronger solvent–adsorbate interactions that further reduce adsorption at higher pH values.^68^ For the CH biochars (BC400 and BC500) and BB500, these combined effects were sufficient to completely inhibit adsorption at pH 12.0.

The constancy of *q*_e_ between pH 2.0 and 10.0 for BB500 suggests that functional groups capable of deprotonation and generating negative charges on this biochar are present in smaller quantities than in the other biochars. Moreover, or most surface groups capable of generating negative charges are deprotonated only above pH 10.0. This is consistent with FTIR results, which showed a decrease in oxygenated functional groups in biochars produced at higher pyrolysis temperatures, as well as with pH_PZC_ results, which indicated a slightly higher pH_PZC_ value for BB500.

**Figure 5:**
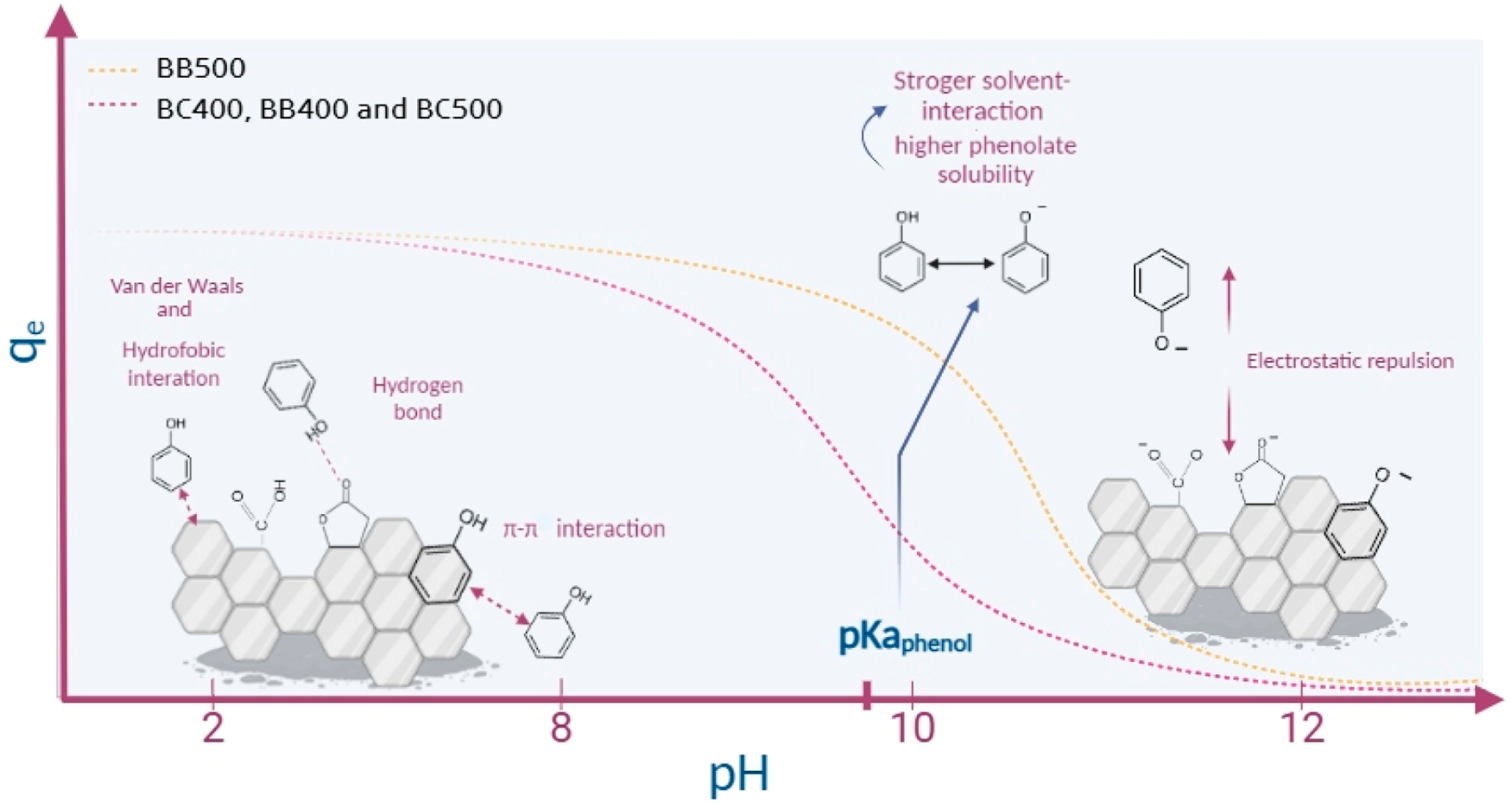
Schematic mechanism of phenol adsorption onto biochars across the pH range.

It should be noted that despite the absence of electrostatic repulsion between the positively charged biochar surface and neutral phenol, the interaction between phenol and the adsorption sites can be hindered at pH 2.0 due to competitive adsorption between phenol and H^+^ ions. This phenomenon can be observed with functional groups like carbonyls, where the presence of H^+^ ions inhibits the formation of electron donor–acceptor complexes with phenol.^67,68^ However, the similar adsorption behavior observed at this low pH and at pH levels near neutrality suggests the existence of π–π interactions between the aromatic group of phenol and aromatic groups on the biochar.^60,69,70^ In addition to hydrophobic interactions, van der Waals forces, and hydrogen bonds, these interactions play a role in determining adsorption in this pH range. Figure 5 illustrates the adsorption mechanisms of the produced biochars across the pH range.

The mechanisms reported in this work are compatible with those described in the literature regarding the adsorption of phenolic compounds on biochar. For example, Mohammed et al. reported that the adsorption of phenol on biochar produced from pine fruit shells involves dispersion interactions as the main contributors to the adsorption process at a pH range between 2.0 and 6.5.^19^ Liu et al., when investigating the removal of nitrophenol from aqueous solutions using biochar derived from pine sawdust, found that the adsorption mechanism involves hydrogen bonding, π–π interactions, and electrostatic interactions.^59^ Nevertheless, it is evident that adsorption on biochar may be influenced by various factors, including not only interactions with specific functional groups but also the porosity of the adsorbent. This latter aspect is discussed in terms of the intraparticle diffusion model in Adsorption Kinetics. Considering the adsorption capacity of phenol by the biochars and the possibility of applying the produced biochars under neutral pH conditions, kinetics and equilibrium studies were performed at pH 7.

**Figure 6:**
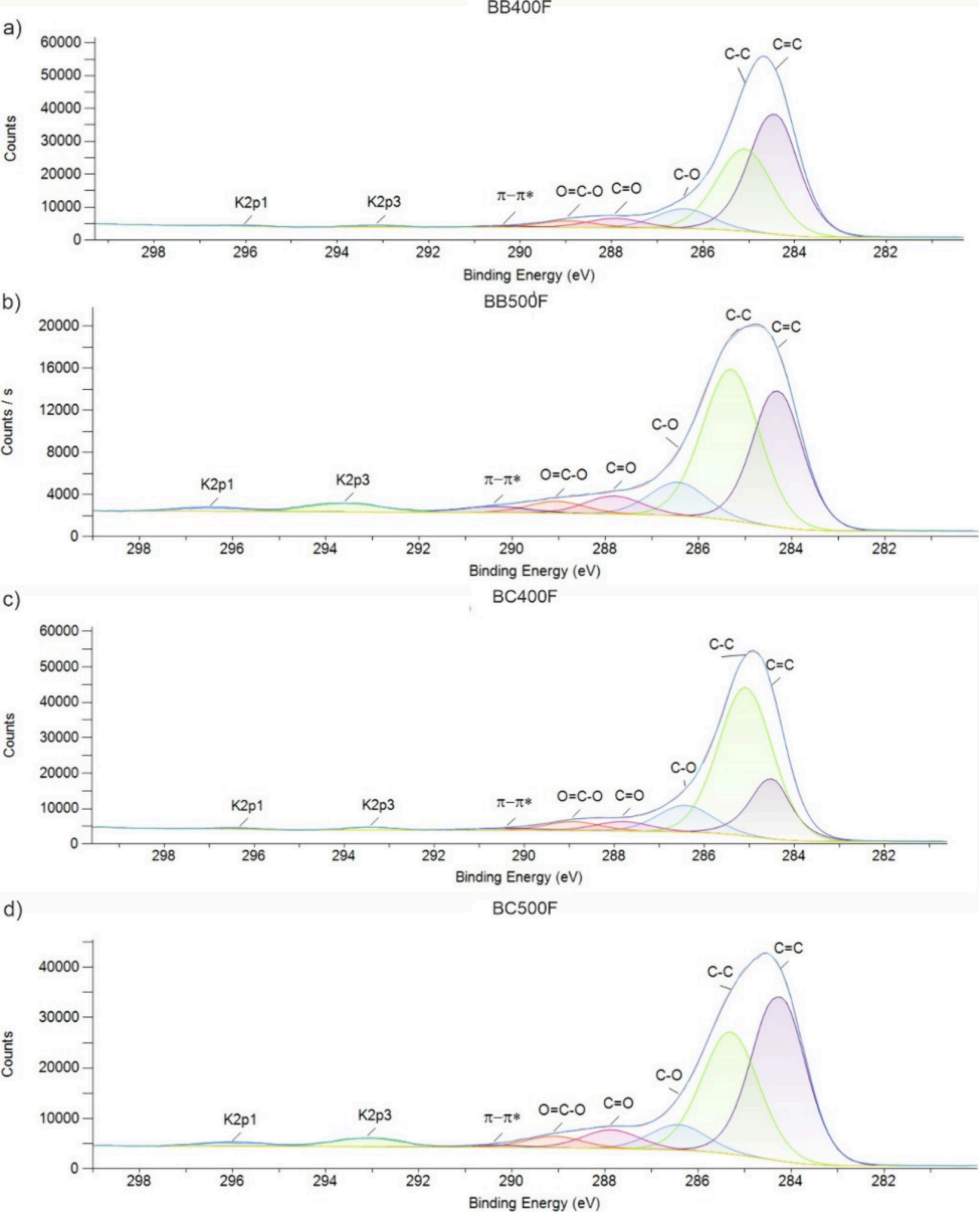
High-resolution C 1s XPS spectra of biochars derived from (a) BB400, (b) BB500, (c) BC400, and (d) BC500 after phenol adsorption.

Regarding phenol adsorption, XPS spectra were acquired from all biochars to confirm the mechanisms of phenol adsorption. Figure 6 shows the XPS spectra and Table 3 shows the atomic percentage of groups after phenol adsorption. In general, a reduction in the C=O (∼287.9 eV) and O=C–O (∼288.8 eV) peaks is observed, suggesting that carbonyl and carboxylate groups on the biochar surface interact with phenol. Additionally, the changes in the π–π* satellite intensities in the phenol-loaded biochars indicates the role of π–π stacking interactions in adsorption.

**Table 3:** Parameters of the PFO, PSO, and Intraparticle Diffusion Models Applied to the Kinetic Data for the Adsorption of Phenol on the BL and CH Biochars^a^

kinetic models					
*Pseudo-first order (PFO)*^b^					
Adsorbent	*q*_*e*,exp_ (mg g^–1^)	*q*_*e*,theo_ (mg g^–1^)^b^	*k*_1_ (h^–1^)^b^	*R*^2^	χ^2^
BC400	5.28	4.46	5.28	0.701	0.16
BC500	5.73	7.40	0.049	0.922	0.35
BB400	6.13	4.79	3.62	0.113	64.6
BB500	6.25	5.07	0.58	0.911	37.2

*Pseudo-second order (PSO)*^b^					
Adsorbent	*q*_*e*,exp_ (mg g^–1^)	*q*_*e*,theo_ (mg g^–1^)^b^	*k*_2_ (g mg^–1^ h^–1^)^b^	*R*^2^	χ^2^
BC400	5.28	4.52	1.89	0.689	0.17
BC500	5.73	10.65	0.0036	0.925	0.33
BB400	6.13	4.86	1.22	0.190	59.0
BB500	6.25	5.59	0.12	0.959	17.1

*Intraparticle diffusion*^c^				
Adsorbent	*q*_*e*,exp_ (mg g^–1^)	*C* (mg g^–1^)^c^	*k_i_* (mg g^–1^ h^–0.5^)^c^	*R*^2^
BC400	6.13	1.85	0.99	0.853
BC500	6.25	0.14	0.77	0.970
BB400	5.28	3.03	0.47	0.828
BB500	5.73	1.55	0.94	0.968

a Adsorption conditions: *C_i_* = 40.0 mg L^–1^, 1.00 g L^–1^ adsorbent, 25.0 °C, pH 7.00, and 150 rpm.

b Parameters obtained using nonlinear regression.

c Parameters from linear regression of the first linear region of *q*_*t*_ versus *t*^1/2^ curves.

Although BB500 exhibits a slightly higher C=O peak intensity when compared with BC500 (as shown in Table 1), this difference is minimal and should not be overstated. Given that both biochars have a negligible specific surface area, the phenol adsorption capacity is primarily determined by surface functional groups and molecular interactions rather than porosity effects. The similar maximum adsorption capacities for both materials (see next sections) further support this conclusion, indicating that π–π* stacking, hydrogen bonding, and hydrophobic interactions control the adsorption mechanism and act similarly for biochar from both biomasses.

#### Adsorption Kinetics

**Figure 7:**
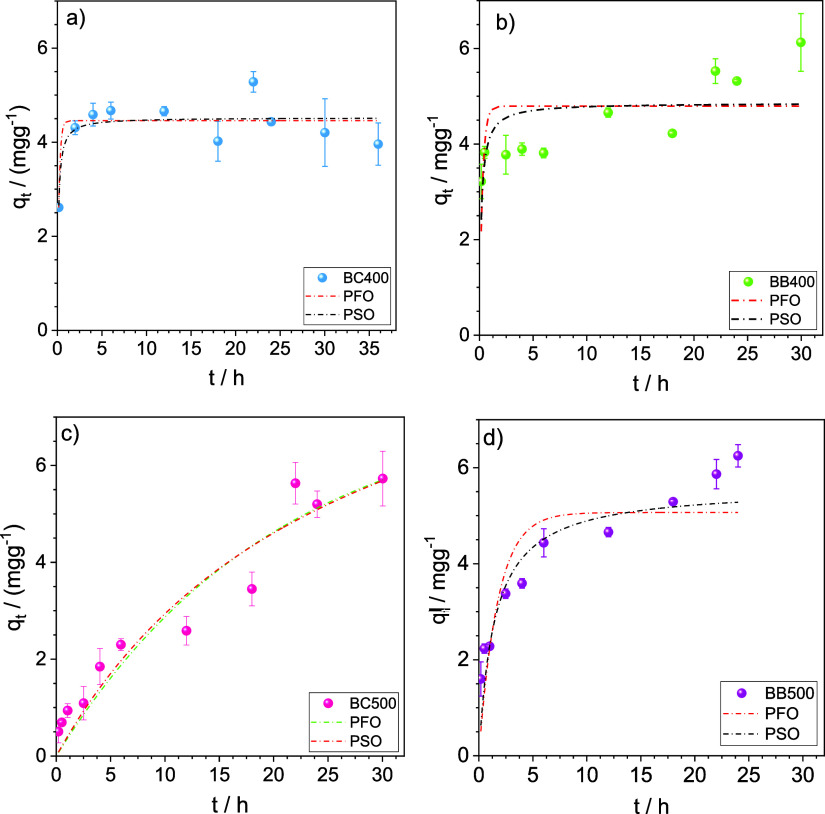
Kinetic curves for the adsorption of phenol on (a) BC400, (b) BB400, (c) BC500, and (d) BB500. Adsorption conditions: *C_i_* = 40 mg L^–1^, 1.00 g L^–1^ of adsorbent, 25.0 °C, pH 7.00, and 150 rpm. Each data point represents the mean of duplicates, with error bars indicating the standard deviation from the mean. For many data points, the standard deviation was smaller than the size of the symbol, making the error bars indistinguishable.

Figure 7 shows the kinetic adsorption curves of phenol on the biochars at pH 7 and 25.0 °C, along with the fits for pseudo-first-order (PFO) and pseudo-second-order (PSO) models. For BC400, phenol transport from the bulk solution to the liquid–solid interface occurred primarily within the first 12 h, reaching steady-state conditions. For BB400, BB500, and BC500, a steady state was reached within 24, 18, and 22 h, respectively.

Although long times were required to reach equilibrium during adsorption, it should be highlighted that for the biochars obtained at the lower pyrolysis temperature (BC400 and BB400), most of the phenol mass was transferred in the first minutes of contact. This was most evident for BC400, where around 90% of the amount adsorbed at equilibrium was reached in the first 30 min of contact. The adsorption rates were slower for the biochars obtained at 500 °C, as indicated by the smaller slopes of the *q*_*t*_*versust* curves for these materials. In this case, more than 10 hours are needed to achieve at least 90% of the adsorption relative to the mass of phenol adsorbed at the steady state.

Table 3 shows the parameters of the models fitted to the kinetic data. Adsorption kinetics on BC400 and BB400 was not well fitted using the PFO and PSO models, with *R*^2^ ≤ 0.701, which is likely associated with the most amount of experimental data close to the steady state, especially for BC400 (fast adsorption). However, these models fitted well to the data from biochar obtained at 500 °C, with the PSO model providing more satisfactory fits (*R*^2^ ≥ 0.925 and χ^2^ ≤ 17.1).

The intraparticle diffusion model was also evaluated to assess the adsorption mechanism. This model is useful for predicting the rate-controlling step.^71^ The parameters derived from the model are shown in Table 2, and the *q*_*t*_ versus *t*^1/2^ curves are shown in Figure S5 (Supporting Information). The linearity of these curves suggests that intraparticle diffusion is involved in the adsorption mechanism for phenol removal. The curves displayed a single linear region with a positive slope, with the equilibrium stage (indicated by a slope near zero) observed only for BC400 and BC500. The intraparticle diffusion model fit was better for biochars produced at 500 °C, suggesting that adsorption for these materials involves sites inside the pores. This indicates that the intraparticle diffusion processes played a more important role in determining the adsorption rates for these biochars. For these materials, the constant *C* exhibited nonzero values for BB500, while for BC500 it was close to zero. This suggests that for BC500 intraparticle diffusion was the primary mechanism influencing the adsorption rate with rapid phenol diffusion from the solution to the adsorbent surface during the initial stages. However, transport to active sites within the pores was the rate-limiting step, which resulted in the prolonged time required to reach equilibrium. This is consistent with the lower diffusion constant (*k_i_*) observed for this biochar, despite the larger average pore size of BC500 compared to BB500 (Table S2). These results suggest that a significant fraction of the adsorption sites in BC500 are likely located within smaller pores. For BB500, the higher *C* value indicates that film diffusion played a important role in the adsorption process, possibly due to a higher content of basic functional groups on the surface (Table 2), which may enhance water structuring on the surface of the material.

#### Adsorption Equilibrium

**Figure 8:**
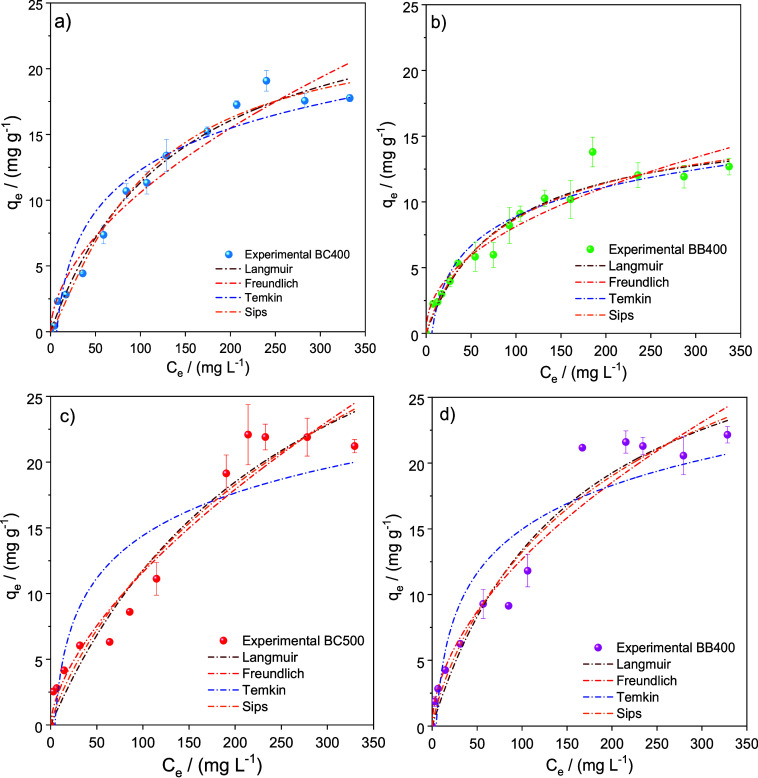
Isotherms for the adsorption of phenol on (a) BC400, (b) BB400, (c) BC500, and (d) BB500. Adsorption conditions: *C_i_* = 40.0 mg L^–1^, 1.00 g L^–1^ of adsorbent, 25.0 °C, pH 7.00, and 150 rpm. Curves obtained from the nonlinear fit of each model are shown. Each data point represents the mean of duplicates, with error bars indicating the standard deviation from the mean. For many data points, the standard deviation was smaller than the size of the symbol, making the error bars indistinguishable.

Figure 8 shows the isotherms and model fits used to evaluate the adsorption of phenol on the biochars produced.

For all studied biochars, the amount of phenol adsorbed increased with the equilibrium concentration, reaching a plateau above a certain *C*_e_ value. Moreover, the *q*_e_ values depended on the biomass type and pyrolysis temperature of the biochar. For the BL biochars, the maximum *q*_e_ values were 13.8 and 21.2 mg g^–1^ for BB400 and BB500, respectively, while for BC400 and BC500, the values were 17.3 and 19.1 mg g^–1^, respectively. Table 4 compares the maximum adsorption capacities obtained in this study with those reported in other studies of phenol adsorption using biochars derived from various feedstocks and pyrolysis conditions.

**Table 4:** Maximum Adsorption Capacities (*q*_max_) for Phenol on Biochars Obtained by Using Different Biomasses and Pyrolysis Conditions

precursor	***q*_max_** (mg g^–1^)	PT (°C)^a^	pore size (Å)	activation	ref.
pine fruit shell	10.73–26.74	350, 450, 550	1070–2360		(19)
sugar cane bagasse	40.33	400, 600, 800	108–224		(72)
kenaf plant	41.1	350, 450, 600, 750	16–27		(12)
banana peel	2.6	550			(20)
oil palm frond	62.89	500	102		(73)
olive oil waste	103.9	550		FeCl_2_	(21)
pomelo peel	24.37	600		FeCl_2_	(22)
*Delonix regia* pot	2.59	375			(74)
cow dung	46.9	550	10–40	KOH	(75)
*Eucalyptus saligna*	76.65	600	76.6	NiCl_2_	(76)
rice straw	107.47	300, 500, 700, 800	37–50	KOH and HCl	(14)
wheat straw	32.79	450–500		Ag nanoparticles	(77)
sewage sludge	34.5	600, 700, 800, 1000	28–192	NaOH, K_2_CO_3_ or CO_2_	(78)
hydrochar from wood feedstock	167	800	43.8	CO_2_	(79)
walnut shell	0.41	500		pristine	(80)
	1.38			Ca-alginate	
nut shell	30.37	600	35–36	KOH	(81)
palm frond	15.93	600	20–500		(82)
orange peel	31.00	700	38.0		(82)
coffee husk	19.1	500	13.24		this work
	17.3	400	17.27		
banana leaf	21.2	500	22.71		this work
	13.8	400	10.27		

a Pyrolysis temperature evaluated in the study.

As observed, the *q*_e_ values vary widely depending on the type of feedstock and the pyrolysis conditions used to produce the biochar. Although biochars generally have a lower adsorption capacity for phenol when compared with activated carbons (Table S1, Supporting Information), they can be a viable alternative for phenol removal from aqueous solutions due to their advantages, particularly lower cost and ease of production. The biochars obtained in this study, in particular, exhibited adsorption capacities comparable to those of other biochars, such as those derived from pomelo peel and pine fruit shell, and higher than those from walnut shell, banana peel, and *Delonix regia* pod. Some of the studied biochars even showed adsorption capacities similar to those of certain activated carbons, indicating the potential of these feedstocks for phenol adsorption. It is worth noting that raw banana residues have also been used for phenolic compound adsorption and showed high performance.^83^ However, over extended storage periods, raw residues are susceptible to degradation and may not be suitable for applications, such as filter development.

In this study, biochars produced at higher pyrolysis temperatures showed greater experimental maximum adsorption capacities (*q*_max_) for phenol. However, it was notable that the adsorptive capacity of CH biochar was less influenced by the pyrolysis temperature, as evidenced by a difference of only 1.86 mg g^–1^ (10%) between the *q*_max_ values for BC400 and BC500. In contrast, for banana leaf biochar, increasing the pyrolysis temperature from 400 to 500 °C led to a 50% increase in the *q*_max_ for phenol. The minimal effect of pyrolysis temperature on the adsorptive capacity of CH biochar may be due to a balance between the loss of basic functional groups and an increase in surface area at higher temperatures. For banana leaf biochar, the substantial increase in maximum adsorption capacity at the higher pyrolysis temperature, despite a reduction in specific surface area, suggests a significant contribution from adsorption sites formed by basic groups, which were enhanced at the higher temperature, in the phenol adsorption process.

Table 5 shows the isotherm parameters for the different isotherm models fitted to the experimental data of phenol adsorption on the biochars.

**Table 5:** Parameters for the Different Isotherm Models Applied to the Isotherm Data for the Adsorption of Phenol on the BL and CH Biochars^a^

Langmuir				
adsorbent	*q*_max_ (mg g^–1^)	*K*_L_ (L mg^–1^)	*R*^2^	χ^2^
BC400	27.5	0.0070	0.984	0.852
BC500	43.0	0.0038	0.950	4.020
BB400	16.5	0.011	0.956	0.860
BB500	34.3	0.0064	0.954	3.390

Freundlich				
adsorbent	*n*	*K*_F_ (L^1/*n*^ mg^1–1/*n*^ g^–1^)	*R*^2^	χ^2^
BC400	1.82	0.85	0.961	2.070
BC500	1.60	0.66	0.949	4.104
BB400	2.20	1.00	0.936	1.256
BB500	1.83	1.03	0.953	3.735

Temkin				
adsorbent	*b*_T_ (J mol^–1^)	*A*_T_ (L mg^–1^)	*R*^2^	χ^2^
BC400	541	0.147	0.938	3.298
BC500	529	0.22	0.828	13.91
BB400	766	0.16	0.937	1.248
BB500	515	0.22	0.880	9.58

Ssips					
adsorbent	*q*_m_ (mg g^–1^)	*K*_s_ (L^*n*_s_^ mg^–*n*_s_^)	*n*_s_	*R*^2^	χ^2^
BC400	23.3	0.0033	1.23	0.986	0.813
BC500	62.3	0.0051	0.83	0.951	4.352
BB400	17.9	0.015	0.90	0.957	0.909
BB500	43.3	0.0094	0.83	0.959	3.628

a Adsorption conditions: 1.00 g L^–1^ adsorbent, 25.0 °C, pH 7.00, and 150 rpm.

The Langmuir isotherm model provided slightly better fits to the experimental data, with *R*^2^ values exceeding 0.95 and χ^2^ values closer to zero. However, considering the *q*_max_values, the model’s estimates were much higher than those observed experimentally under surface saturation conditions (see Table 3), except for BB400. Regarding the Sips model, it combines the Langmuir and Freundlich equations and approaches the Langmuir model when *n*_s_ = 1. This model shows a quality similar to Langmuir’s, but with *n*_s_ values smaller than unity, corroborating the tendency of the system toward surface heterogeneity. In this context, it is noteworthy that the Freundlich model also provided a good fit to the data, with fitted curves from the Langmuir and Freundlich models closely overlapping in Figure 6. The Freundlich model better aligns with the possibility of interactions between phenol and various adsorption sites on the surface (Figure 4), suggesting that heterogeneous sites were involved in the process, with variable interaction intensities between phenol and these sites.^84^ This heterogeneity, which is commonly observed in the structures of charcoal and activated carbons, is related to the variability of the functional groups present,^85^ as shown for the biochars obtained in this work. The higher *n* value for BB400 suggested that the interactions between phenol and the adsorption sites were on average more intense for this material. This implies that carbonyl groups or other oxygenated groups retained at this biochar produced at lower pyrolysis temperature, as indicated by the FTIR data, likely played a key role in the phenol adsorption process. Naturally, this material did not have the highest maximum adsorption capacity, as this parameter is determined by the number of available sites, which depends on other factors, including morphological aspects.

To verify the hypothesis of formation of multilayer adsorption and/or pore filling by phenol, the maximum experimental *q*_e_ values obtained in this work were expressed in mg m^–2^ using BET-specific surface area data (Table S2, Supporting Information). The higher obtained values were around 8 mg m^–2^ (corresponding to approximately 51 molecules of phenol per nm^2^) for BC400 and BB500. Phenol has a topological polar surface area equals to 2.02 nm,^86^ suggesting that most of the phenol molecules are not directly interacting with the sites of the adsorbent. Analyzing these results, the materials with the higher number of molecules adsorbed per nm^2^ (BC400 and BB500) were also those with the higher number of both acid and basic functions, and, especially BC400, was that with the smaller pore volume (Table S2, Supporting Information). This shows that despite multilayer adsorption being able to make an important contribution to phenol adsorption, the magnitude of specific interactions between phenol and the surface sites determined the order of adsorption capacity of the evaluated materials.

## Conclusions

Biochars derived from coffee husks and banana leaves at various pyrolysis temperatures exhibited distinct capacities for phenol retention in aqueous solutions. The surface basic group content was the main factor influencing the *q*_e_ values. While an increase in the pyrolysis temperature resulted in an approximate 10% increase in the maximum adsorption capacity of coffee husk biochars, the increase was more substantial (around 50%) for banana leaf biochars. This suggests that pristine biochar derived from coffee husks at lower temperatures is less sensitive in terms of phenol adsorption capacity. Despite differences in phenol retention, the studied biochars showed similar adsorption mechanisms, involving pore filling and/or multilayer adsorption at heterogeneous sites. For materials produced at higher temperatures, intrapore diffusion emerged as the dominant mechanism influencing adsorption. The results demonstrated the potential of biochars to adsorb phenol across a broad pH range (2–10), which covers typical wastewater conditions. Within this range, interactions such as π–π stacking, hydrophobic interactions, and hydrogen bonding were predominant. The *q*_e_ values achieved in this study for biochars derived from coffee husks and banana leaves exceeded those reported for other biomass sources, such as banana peels, walnut shells, palm fronds, and pine fruit shells. This approach underscores the potential of repurposing abundant, low-value organic waste for the removal of critical pollutants. Future research should explore chemical activation and/or surface functinalization of biochars from these materials to further enhance their adsorption performance.

## Methodology

### Reagents and Biomass Wastes

The reagents used in this work are shown in Table 6. Leaves of the banana species *Musa acuminata* ‘Cavendish’ were collected in the city of Ouro Preto (Minas Gerais State, Brazil). The husks of*Coffea arabica* were collected at Fazenda Limeira, in the city of Nepomuceno (Minas Gerais State, Brazil). Deionized water was used in all of the experiments.

**Table 6:** Technical Information on the Reagents Used in This Work

reagent	formula	CAS	teor/concentration	supplier
phenol	C_6_H_6_O	108-95-2	≥99%	Sigma-Aldrich
phenol standard solution	C_6_H_6_O		1000.2 ± 6 mg L^–1^	SpecSol
potassium acid phosphate	K_2_HPO_4_	7758-11-4	98.0%	Synth
potassium dihydrogen phosphate	KH_2_PO_4_	7778-77-0	99.0%	Synth
phosphoric acid	H_3_PO_4_	7664-38-2	85%	Synth
sodium hydroxide	NaOH	1310-73-2	98.0%	Synth
potassium biphthalate	C_8_H_5_KO_4_	877-24-7	99.5%	Isofar
hydrochloric acid	HCl	7647-01-0	37%	Neon
sodium chloride	NaCl	7647-14-5	analytical grade	Reagen

### Biomass Preparation and Biochar Production

The biochar was prepared according to de Castro et al.^87^ The biomass samples were dried at 45 °C for 24 h, followed by grinding in a knife mill (MAO048, Marconi). A portion of 32 g of the dried and ground biomass was pyrolyzed into a tubular oven with three independent heating zones (Model FT-HI, EDG). The samples were heated using a temperature ramp of 10 °C min^–1^, under a flow of argon at 400 mL min^–1^. The final pyrolysis temperature was maintained for 2 h and then cooled naturally to room temperature. The final pyrolysis temperature was 400 or 500 °C, producing biochars BB400 and BB500 from BL, and BC400 and BC500 from CH. The biochars were sieved to a maximum particle size of 100 mesh.

### Characterization of the Biochars

The methodologies used to determine the point of zero charge (pH_PZC_) values and the number of acidic and basic groups of the biochars are presented in the Supporting Information.

#### Determination of Surface Area, Pore Size, and Pore Volume

Isotherms of adsorption/desorption of N_2_, at 77 K, were obtained for the previously degassed (at 80 °C for 5 h) biochars by using a Micromeritics ASAP instrument. The pore volumes and sizes were obtained by density functional theory (DFT).

#### Raman Spectroscopy

Raman spectra of the biochars were acquired by using a Horiba LabRAM HR Evolution spectrometer equipped with a 532 nm laser and 50× objective lens. The spectra were recorded in the range between 300 and 3000 cm^–1^. The crystallite size (*L*_a_) was calculated as described by Ribeiro-Soares et al.^88^

#### Infrared Spectroscopy

Infrared spectra of the biochar samples were acquired using a Varian 600-IR Series spectrometer operated in an attenuated total reflection mode. The spectra were obtained between 500 and 4000 cm^–1^, at a resolution of 4 cm^–1^, with 32 scans.

#### SEM and EDS

The morphology and the elementar composition of the produced biochars were analyzed using a scanning electron microscope—STEM-FEG ultrahigh-resolution and field-free (TESCAN VEGA 3). This instrument was equipped with an X-ray energy-dispersive microanalysis system (Oxford Instruments, X-act), with analyses conducted at 20 keV. For sample preparation, an amount of biochar was deposited on an aluminum support covered with double-sided carbon tape. Subsequently, a vacuum treatment (Quorum Q150R ES) was performed to remove impurities and the samples received a carbon bath to increase their conductivity.

#### X-ray Photoelectron Spectroscopy

The XPS analyses were performed using an X-ray photoelectron spectrometer from Thermo Scientific, with an excitation of 1.4866 eV (aluminum K-α line) and a spot size of 650 μm. Band pass energies of 100 and 20 eV were used for survey and high-resolution spectral acquisition of *C*, respectively. In the initial phase, preliminary measurements were conducted at three different points on each sample to determine the optimal parameters and evaluate the total acquisition time. After analyzing and confirming the homogeneity of the requested spectra, one point from each sample (those with the most intense spectra) was selected for the final measurements. The parameters used were 10 scans with 100 ms for survey acquisition and 10 scans with 200 ms for high-resolution acquisition, and the spectra were acquired using the flood gun functionality for charge compensation. For the deconvolution of the peaks, all components used were symmetric signals, except for the C 1s C=C peaks. Reference data were extracted from the literature and used here.^89^

### Adsorption Assays

The adsorption experiments were conducted based on previous work developed by our research group, with some modifications.^90^

#### Effect of pH

Solutions of 0.100 mol L^–1^ phosphate buffer were prepared at pH 2.00, 7.00, 10.00, and 12.00. These solutions were used as solvents to prepare solutions of phenol at 40.0 mg L^–1^. At each pH, 20.00 mL of phenol solution were added to an Erlenmeyer flask containing 0.0200 g of biochar, weighed out using an analytical balance (Model ATX224, Shimadzu). The flasks were agitated for 22 h in a shaker–incubator (Model TE-424, Tecnal), at 25.0 °C and 150 rpm, and after this time, the systems were transferred to 50 mL centrifuge tubes to centrifugation (MOD 280 centrifuge, FANEM) at 3500 rpm. Then, the supernatants were collected for determination of the phenol concentrations by UV–vis spectroscopy (Model UV-1800, Shimadzu) at wavelengths of 270 nm (for pH 2.00, 7.00, and 10.00) or 287 nm (for pH 12.00).

#### Adsorption Kinetics

Adsorption kinetics experiments were performed at pH 7.00, and the experiments were performed as described inEffect of pH. Summarily, 20.00 mL of 40 mg L^–1^ phenol solution and 0.0200 g of biochar were used for each experimental point, i.e., for each time evaluated (0.5; 1; 2; 4; 6; 10; 14; 18; 22; 24; 28; and 30 h).

#### Adsorption Isotherms

Adsorption isotherms of phenol on the biochars were obtained at pH 7.00, and the experiments were performed as described in Effect of pH. Particularly, the concentration of phenol solution mixed with 0.0200 g of biochar for each experimental point varied in the range of 2.5–350 mg L^–1^. The systems were placed in a shaker–incubator and kept at 150 rpm and 25.0 °C until the equilibrium was reached.

#### Calculation of the Amounts of Phenol Adsorbed

The quantities of phenol adsorbed for each adsorption system (sections Effect of pH to Adsorption Isotherms) were determined by mass balance, according to eq 1(84):
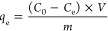
1where *q*_e_ is the amount of phenol adsorbed per unit mass of adsorbent; *V* is the volume of the solution; *m* is the mass of biochar; and *C*_0_ and *C*_e_ are the initial and equilibrium concentrations of adsorbate in the phase where it was present in the dissolved form, respectively. In the kinetic studies, *q*_e_ and *C*_e_ were substituted by *q*_*t*_ and *C*_*t*_, respectively, denoting the properties obtained for each time investigated. All the analyses were performed in duplicate.

#### Adsorption Modeling

The adsorption kinetics curves were fitted using the pseudo-first-order (PFO), pseudo-second-order (PSO), and intraparticle diffusion models. For the PFO model, the rate of adsorption of the adsorbate is described by eq 2(59):
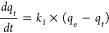
2where *q*_e_ and *q*_*t*_ are the quantities of phenol adsorbed at equilibrium and at time *t*, respectively, and *k*_1_ is the pseudo-first order rate constant.

The PSO model, which can be used to fit data for a wider adsorption time range, is given by eq 3(59);
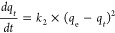
3where *q*_e_ and *q*_*t*_ have the same significance as in eq 2 and *k*_2_ is the pseudo-second order rate constant. The model fit parameters were determined by nonlinear regression using the integrated forms of eqs 2 and 3.

The intraparticle diffusion model assumes that diffusion through the liquid film that solvates the adsorbate is negligible; therefore, the adsorption rate is determined by the intraparticle diffusion process. This model can be described by eq 4:
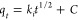
4where *k*_*i*_ is the diffusion constant, *C* is a constant related to the thickness of the liquid film, and *q*_*t*_ is the quantity adsorbed at time *t*. The parameters *k*_*i*_ and *C* were obtained by the slope and intercept, respectively, of the linear regression from the graphs of *q*_*t*_ versus *t*^1/2^.

The isotherms obtained were fitted by using the models summarized in Table 7. Parameters were obtained using nonlinear regression. The qualities of the model fits were evaluated by using the coefficient of determination (*R*^2^) and χ^2^.

**Table 7:** Adsorption Isotherm Models Applied to the Studies of Phenol Adsorption on the Biochars

**model**	**equation**	**fitting parameters**		**ref.**
Langmuir		*q*_max_	maximum adsorption capacity (mg g^–1^)	(9)
		*K*_L_	Langmuir constant (L mg^–1^)	
Freundlich		*K*_F_	Freundlich constant (L^1/*n*^ mg^1–1/*n*^ g^–1^)	(94)
		*n*	intensity of adsorbent–adsorbate interactions	
Temkin		*b*_T_	Temkin constant (J mol^–1^)	(94)
		*A*_T_	equilibrium binding constant (L mg^–1^)	
Sips		*q*_m_	maximum adsorbed amount (mg g^–1^)	(9)
		*K*_s_	Sips constant (*L*^*n*_s_^ mg^–*n*_s_^)	
		*n*_s_	Sips constant	

## Data Availability

The authors confirm that the data supporting the findings of this study are available within the article [and/or] its Supporting Information.

## Supporting Information Available

Methodologies used for the characterization of biochars including determining the point of zero charge, quantifying acidic and basic functional groups, and comparing the adsorption capacities of activated carbons reported in the literature; analyses of surface area, Raman spectra, crystallite size, point of zero charge, elemental distribution via EDS, and kinetic modeling of intraparticle diffusion (PDF)
