# Supplementary material for: Mechanisms of Phenol Adsorption on Banana Leaves and Coffee Husk Biochars
Source: ACS Omega. 2025 Apr 16;10(16):15989–6005. doi: 10.1021/acsomega.4c07665 (PMC12044448; doi:10.1021/acsomega.4c07665)
Supplement: Supplementary file 1 — ao4c07665_si_001.pdf [file ao4c07665_si_001.pdf]

Mechanisms of the Phenol adsorption on banana leaves and coffee husk biochars

Melany Alejandra Ruiz Lopez[a], Guilherme Max Dias Ferreira[b*], Matheus Torres Duarte Figueiredo[a], Gabriel Max Dias Ferreira[a], José Romão Franca[c], Evanise da Silva Penido[b], Jenaina Ribeiro Soares[c], Raphael Longuinhos Monteiro Lobato[c] and Aparecida Barbosa Mageste[a,*]

[a]*Laboratory of Physical Chemistry and Environmental Chemistry, Department of Chemistry, Federal University of Ouro Preto, Campus Morro do Cruzeiro, Ouro Preto, MG, 35400-000, Brazil.*

[b]*Department of Chemistry, Institute of Natural Sciences, Federal University of Lavras, Campus Universitário, Lavras, PO Box 3037, Minas Gerais, Brazil.*

[c]*Department of Physics, Federal University of Lavras, Campus Universitário, Lavras, Minas Gerais, Brazil.*

* Corresponding authors.

aparecida.mageste@ufop.edu.br (A.B. Mageste)

guilherme.ferreira@ufla.br (G.M.D. Ferreira)

Methodology for determination of point of zero charge (PZC) of the biochars

The PZC was estimated according to the methodology by [1]. For this, masses of 0.0200 g of biochar were mixed with 25.00 mL volumes of 0.0100 mol L^{-1} NaCl solution that had been previously adjusted to different pH values with solutions of HCl or NaOH. The systems were agitated for 24 h in a shaker-incubator (Model TE-424, TECNAL), at 25.0 ºC and 150 rpm. The suspensions were centrifuged (Model MOD 280, FANEM) for 15 min at 3000 rpm, followed by measurement of the pH of the supernatant. Each analysis was performed in duplicate.

Methodology for determination of the content of acidic and basic groups on biochars

The content of acidic and basic groups on the surfaces of the biochars were determined according to [2]. Briefly, a mass of 0.1000 g of biochar was added to a jacketed beaker containing an aqueous 0.0085 mol L^{-1} HCl solution. The suspension was agitated for 1 h, at 25.0 ºC, until a constant conductivity (Model CG 1800, Gehaka). The suspension was then titrated with consecutive 0.200 mL additions of 0.1682 mol L^{-1} NaOH solution. Titration was also performed in the absence of biochar, following the same procedure.

For each composition, the corrected conductivity (K_c) was calculated using Eq. 1:

$$K_c = K\frac{(V_i+V)}{V_i} \tag{1}$$

where, K is the measured conductivity (µS cm^{-1}); V_i is the initial volume of the system titrated (mL); and V is the volume of NaOH solution added (mL). The quantities of acidic and basic functions (in mmol/g) on the surfaces of the biochars could be obtained using Eqs. 2 and 3:

$$n_{basic\ functions} = \frac{C_{NaOH}(V_0-V_1)}{m} \tag{2}$$

$$n_{acidic\ functions} = \frac{C_{NaOH}(V_2-V_1)}{m} \tag{3}$$

where, C_{NaOH} is the concentration of the NaOH titrant; V_0 is the equivalence point volume for titration of the blank; V_1 and V_2 are the first and second equivalence point volumes, respectively, in the titration curve for the suspension containing biochar; and m is the mass of biochar.

Table S1. Capacities of Adsorption of Activated Carbon for Phenol Adsorption.

Precursor	q_e (mg/g)	PT (°C) [a]	Pore size (Å)	Activation	Ref.
Coconut shell	27.53-30.35	500	-	KOH	[3]
Rice husk	201.00	450, 750	>1000	KOH	[4]
Coconut shell	300	400, 700, 800, 900	480	NaOH	[5]
Cow-dung	518.89	500	10-40	KOH	[6]
Lead ferrite activated carbon	145.71	700	40-119	Fe^{3+}, Pb^{2+}	[7]
Sugarcane bagasse and sawdust	158.9	750	-	KOH, Ar, and K_2CO_3	[8]
Food residues	14.61	300, 500, 700, 900	-	HNO_3	[9]
Peanut husk	29.01	600°C	40.1	CO_2	[10]
Exhausted coffee	137.91	450, 600	22.3-33	$ZnCl_2$	[11]
Commercial activated carbon	132.3-500	-	49.8	-	[12, 13]

[a] Pyrolysis temperature

Table S2. Textural Analysis of the Biochars Obtained from Coffee Husks and Banana Leaves.

Adsorbent	Specific surface area (m^2/g)	Pore volume (cc/g)	Pore size average (Å)
BC400	2.095	0.003727	27.19
BC500	3.633	0.004591	22.71
BB400	3.295	0.009231	14.48
BB500	2.598	0.005316	13.24

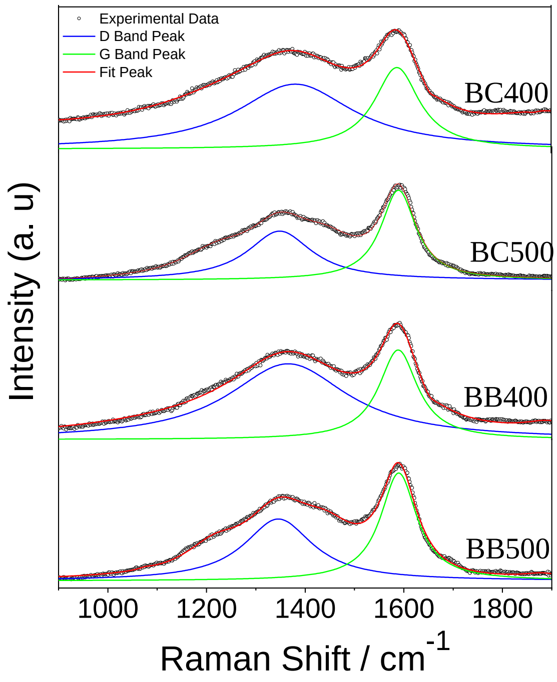

Figure S1. Raman spectra of the biochars adjusted with Lorentz functions

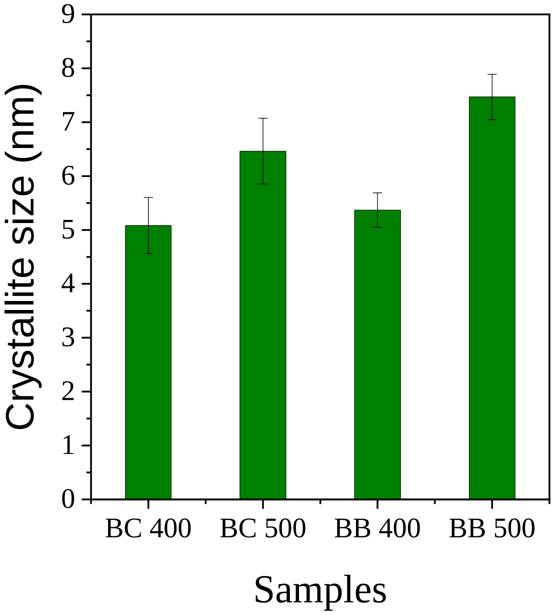

Figure S2. Values of L_a estimated from Raman spectra data

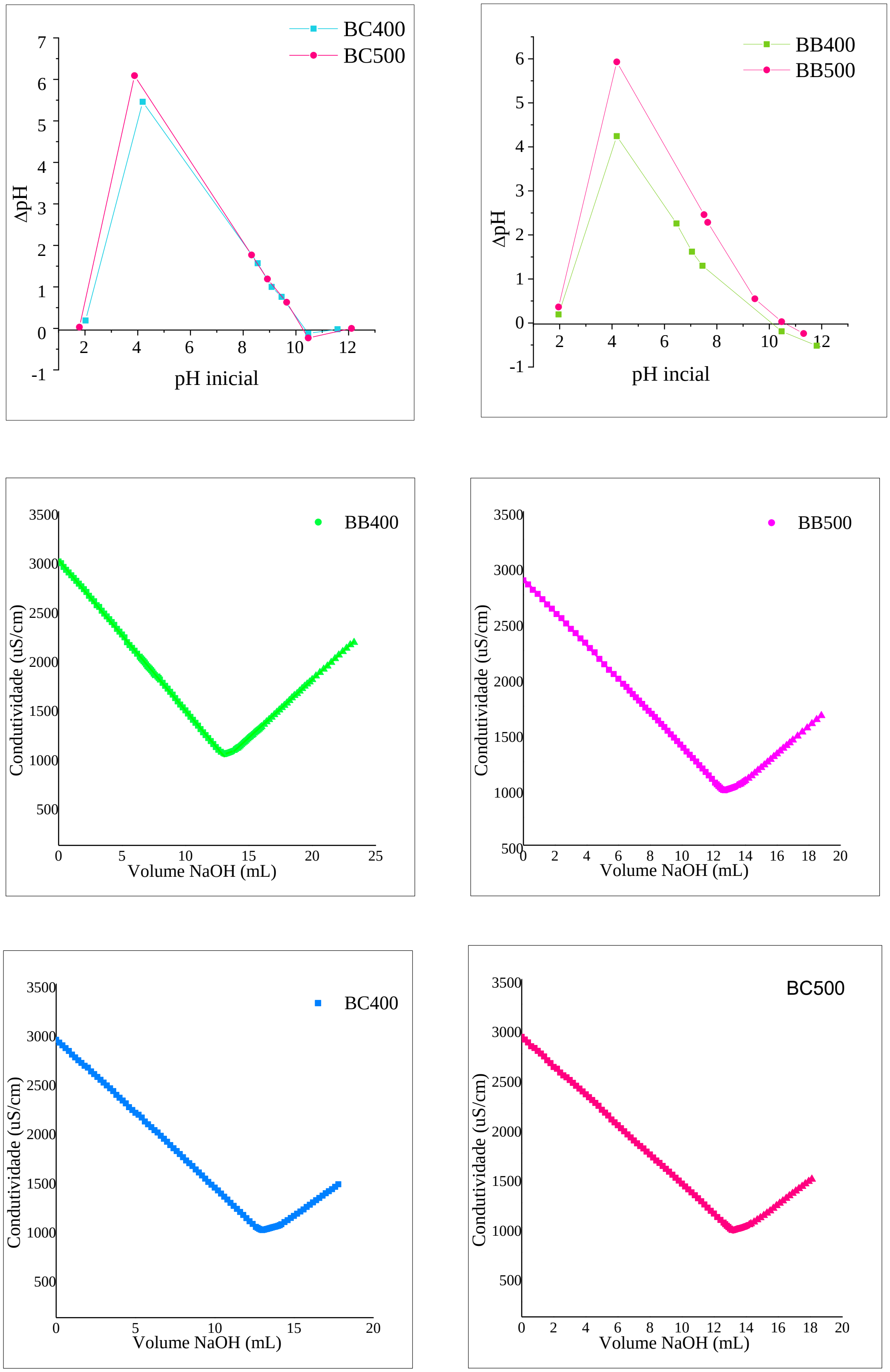

Figure S3. PZC determination and conductimetric titration of coffee husks (BC) and banana leaves (BB).

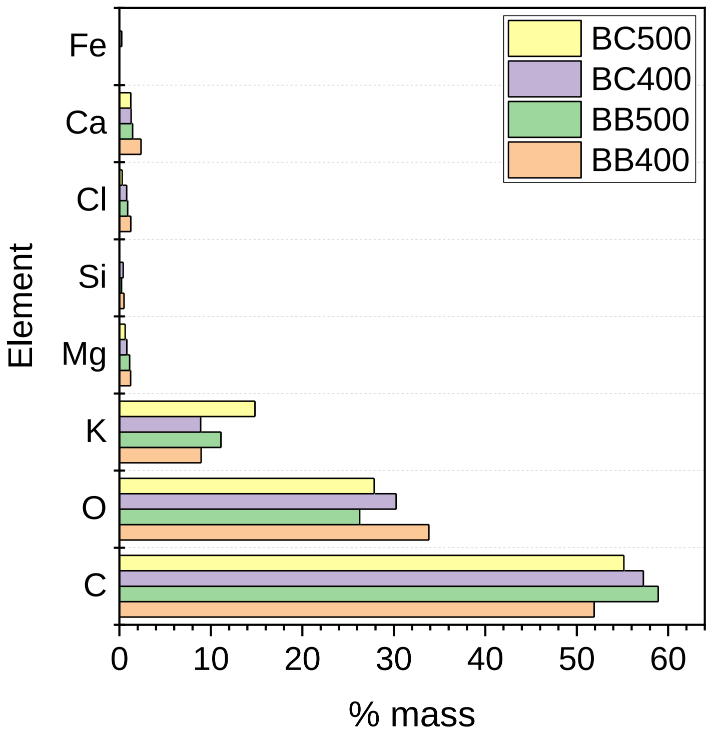

Figure S4. Elements distribution provided by EDS.

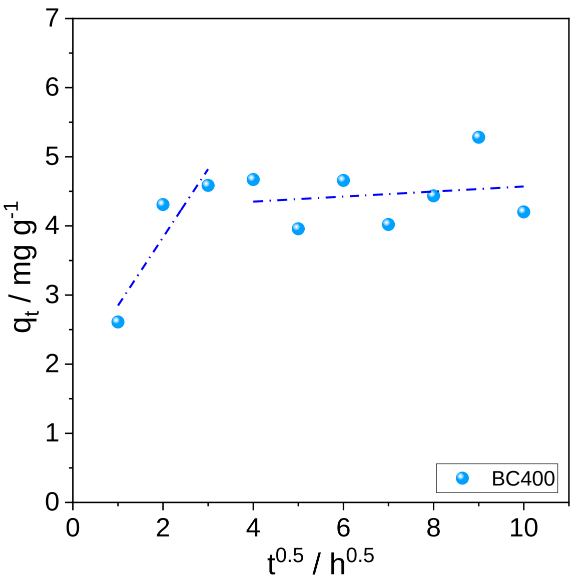

BC400
q_t / mg g^{-1}
$t^{0.5}$ / $h^{0.5}$

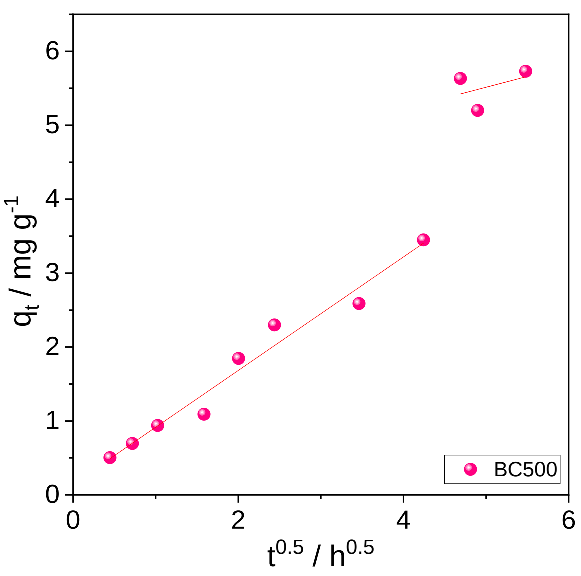

BC500
q_t / mg g^{-1}
$t^{0.5}$ / $h^{0.5}$

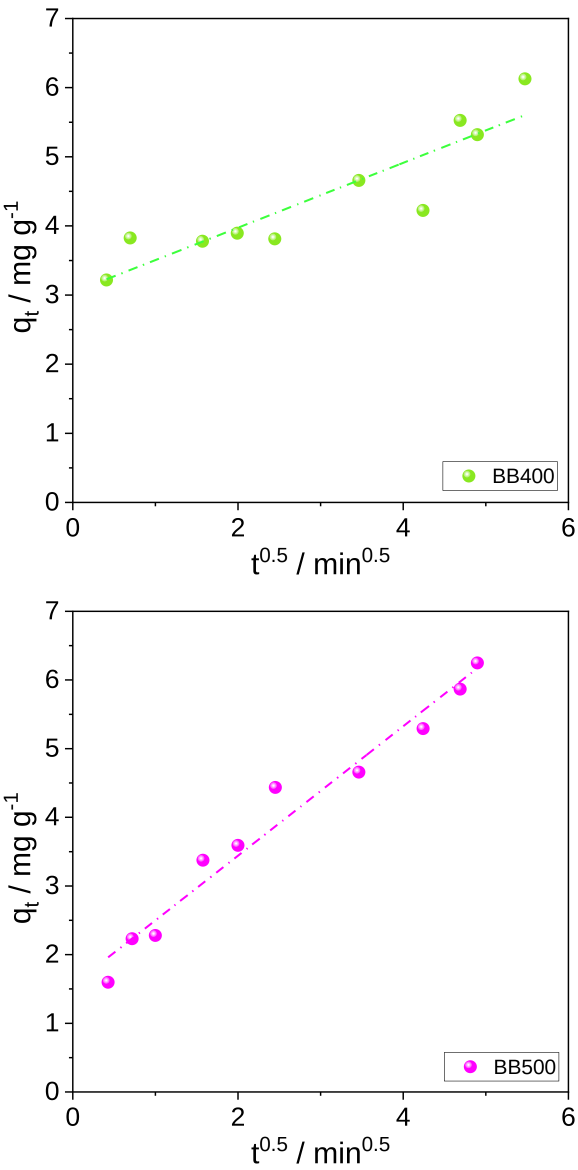

Figure S5. Kinetic data fitted to the intraparticle diffusion model for phenol adsorption on biochars.

References

1) Jesus,A.S.; Ferreira,G.M.D.; Ferreira,G.M.D.; Souza,T.F.; Siqueira,K.P.F.; Nogueira,A.E.; Mageste,A.B. Composite of Organo LDH and biochar for diclofenac sodium removal from aqueous solutions.Mater.Chem.Phys. 2024, 328, No.129919.

2) Pereira, A.R.; Soares, L.C.; Teodoro, F.S.; Elias, M.M.C.; Ferreira, G.M.D.; Savedra, R.M.L.; Siqueira, M.F.; Martineuau-Corcos, C., da Silva, L.H.M.; Prim, D.; Gurgel, L.V.A.: Aminated cellulose as a versatile adsorbent for batch removal of As(V) and Cu(II) from mono- and multicomponent aqueous solutions. J Colloid Interface Sci. 2020, 576, 158-75.

3) Moreno-Piraján, J.C.; Giraldo, L.; Gonzalez, J.F. Adsorción de Fenol en soluciones acuosas empleando monolitos de carbón activado de cáscara de Coco: isotermas y cinéticas de adsorción. Afinidad LXVIII. 2011, 68, 290-95.

4) Shen, Y. Rice Husk-Derived Activated Carbons for Adsorption of Phenolic Compounds in Water. Global Challenges. 2018, 2, No.1800043.

5) Li, M.; Wang, Y.; Liu, Y.; Wang, H.; Song, H.: Preparation of active carbon through one-step NaOH activation of coconut shell biomass for phenolic wastewater treatment. Rev. Chem. Intermed. 2022, 48, 1665-84.

6) Jain, M.; Khan, S.A.; Sahoo, A.; Dubey, P.; Pant, K.K.; Ziora, Z.M.; Blaskovich, M.A.T. Statistical evaluation of cow-dung derived activated biochar for phenol adsorption: Adsorption isotherms, kinetics, and thermodynamic studies. Bioresour Technol. 352, 2022, No.127030.

7) Allahkarami, E.; Monfared, A. D.; Silva, L.F.O.; Dotto, G.L. Lead ferrite-activated carbon magnetic composite for efficient removal of phenol from aqueous solutions: synthesis, characterization, and adsorption studies. Scientific Reports. 2022, 12, No.10718.

8) El-Bery, H.M.; Saleh, M.; El-Gendy, R.A.; Saleh, M.R.; Thabet, S.M.: High adsorption capacity of phenol and methylene blue using activated carbon derived from lignocellulosic agriculture wastes. Sci. Rep. 2022, 123, No.5499.

9) Lee, C.; Hong, S.; Hong, S.; Choi, J.; Park; S. Production of Biochar from Food Waste and its Application for Phenol Removal from Aqueous Solution. Water Air Soil Poll. 2019, 230, No. 70.

10) Silva, T., Barbosa, C., Gama, B., Nascimento, G., Duarte, M.: Agregação de valor à resíduo agroindustrial: Remoção de fenol utilizando adsorvente preparado a partir de casca de amendoim. Revista Matéria. No.e-11947.

11) Garza, M. L.L.D.L. Determinación de las Condiciones Óptimas para la Producción de Carbón Activado Granular a Partir de Residuos de Café para la Eliminación de Fenol en Solución Acuosa, 2014.

12) Zango, Z.U.; Sambudi, N.S; Jumbri, K.; Ramli, A.; Bakar, N.H.H.A; Saad, B.; Rozaini, M.N.H.; Isiyaka, H.A.; Osman, A. M.; Sulieman, A. An Overview and Evaluation of Highly Porous

Adsorbent Materials for Polycyclic Aromatic Hydrocarbons and Phenols Removal from Wastewater. Water 2020, 12, No.2921.

13) Hwang, H.; Sahin, O.; Choi, J.W. Manufacturing a super-active carbon using fast pyrolysis char from biomass and correlation study on structural features and phenol adsorption. RSC Adv. 2017, RSC Adv., 2017,7, 42192-42202.
